# Investigating bacteria-induced inflammatory responses using novel endometrial epithelial gland organoid models

**DOI:** 10.3389/frph.2024.1490520

**Published:** 2024-11-12

**Authors:** Xin Zhang, Li Zhang, Ting Li, Zhan Zhang, Xiang Shang, Huihui Bai, Yong Liu, Xiaonan Zong, Chenguang Shang, Dan Song, Xu Zhang, Linyuan Fan, Zhaohui Liu

**Affiliations:** ^1^ Department of Gynecology, Beijing Obstetrics and Gynecology Hospital, Capital Medical University, Beijing Maternal and Child Health Care Hospital, Beijing, China; ^2^ Department of Clinical Laboratory, Beijing Obstetrics and Gynecology Hospital, Capital Medical University, Beijing Maternal and Child Health Care Hospital, Beijing, China; ^3^ Department of Gynecologic Oncology, Beijing Obstetrics and Gynecology Hospital, Capital Medical University, Beijing, China; ^4^ Laboratory of Electron Microscopy, Pathological Center, Peking University First Hospital, Beijing, China

**Keywords:** endometrial epithelial gland organoids, infection model, endometrial receptivity, hormone responsiveness, MAPK signaling pathway

## Abstract

**Introduction:**

The endometrium plays a crucial role in early human pregnancy, particularly in embryo implantation, survival, and growth. However, invasion and infection by pathogens can lead to endometritis, infertility, and poor reproductive outcomes. Understanding the mechanisms of endometritis and its impact on fertility remains limited. An infection model using patient-derived endometrial epithelial gland organoids (EEGOs) was established to advance *in vitro* studies on endometritis and related infertility.

**Methods:**

An EEGOs infection model was constructed and characterized from human endometrium, treating the organoids with estrogen and progesterone to observe changes in the proliferative and secretory phases. The organoids were infected with *E. coli*, and the release of inflammatory cytokines in the supernatant was detected using ELISA. RNA-seq was employed to analyze the differences before and after *E. coli* treatment, and differential gene mRNA expression was validated using real-time quantitative PCR. Additionally, the effect of E2 in alleviating inflammation was assessed through markers of receptivity (PAEP, LIF, ITGβ), proliferation (Ki67), and barrier repair (ZO-1).

**Results:**

The constructed human EEGOs exhibited long-term expansion capability, genetic stability, and characteristic hormonal responses, strongly expressing epithelial markers (MUC1, E-Cadherin). After *E. coli* infection, the expression levels of inflammatory cytokines TNF-α, IL-8, and IFN-γ increased significantly (*P* < 0.05). RNA-seq indicated that the MAPK signaling pathway was activated post-infection, with increased expression levels of heat shock proteins and transcription factor mRNA. E2 treatment post-infection significantly decreased the mRNA expression of inflammatory genes IL-1β, IL8, IL6 and TNF-α compared to the *E. coli* infected group (*P* < 0.05). Additionally, the expression of genes related to receptivity, proliferation, and barrier repair was enhanced in the E2-treated organoids.

**Conclusions:**

Our findings demonstrate that patient-derived EEGOs are responsive to bacterial infection and are effective models for studying host-pathogen interactions in bacterial infections. These organoids revealed the anti-inflammatory potential of E2 in alleviating *E. coli*-induced inflammation, providing insights into the mechanisms of endometritis and its impact on infertility. The study supports the use of EEGOs as valuable tools for understanding endometrial health and developing targeted treatments.

## 1 Introduction

Embryo implantation constitutes a complex and precise physiological process essential for both natural conception and assisted reproductive technology (ART), involving synchronized changes between the embryo and the endometrium (1). This process involves the recognition, positioning, and attachment of a free blastocyst to the endometrial epithelial cells, followed by the penetration of trophoblast cells through the endometrial epithelium and invasion into the underlying stroma. The invasion continues as the trophoblast cells breach the basal membrane, reach the spiral arteries, and contribute to placental formation, establishing a stable and functional maternal-fetal interface (2). Studies indicate that implantation failure primarily correlates with maternal, embryonic, genetic, and environmental factors. Even after identifying potential causes and implementing interventions aimed at improving implantation rates, some cases remain unexplained, indicating factors yet to be understood (3).

Endometritis represents an infectious and inflammatory condition affecting the endometrium (4). Chronic endometritis (CE) is characterized by superficial endometrial edema, increased stromal cell densities, maturation of the epithelium-stroma interface, and infiltration by endometrial stromal cells (5). Commonly detected microorganisms in endometritis include bacteria such as Streptococcus, *E. coli,* Enterococcus faecalis, and Staphylococcus (6). *E. coli* emerges as a primary bacterial pathogen responsible for endometritis (7). Endometritis negatively impacts endometrial receptivity, thereby contributing to infertility (8).

The study of endometrial models has progressed from *in vitro* cell lines to animal models. *In vitro* 2D culture restricts these cells from achieving cell-cell interactions, a crucial feature of the complex *in vivo* microenvironment (9). Moreover, using non-human primates in research is challenging due to high costs, specialized resources, and ethical controversies. There are significant anatomical and physiological differences between the female reproductive tracts of humans and mice, which require careful consideration in research. While mice share the hemochorial type of placentation with humans, their intrauterine trophoblast invasion is considerably shallower. Additionally, mice normally have multiple pregnancy, which further distinguishes them from human reproductive biology. Recently, substantial progress has been made in generating and applying self-organizing three-dimensional structures known as organoids (10). After enzymatic digestion, endometrial tissue matures into hollow spherical organoids in specific media, maintaining genetic stability during cell division and differentiation. They also recover secretory features post-cryopreservation (11). Additionally, epithelial cells of endometrial epithelial gland organoids (EEGOs) exhibit positive responses to estrogen and progesterone, paving the way for research on endometrial-related disorders (12). Traditional 2D endometrial cell cultures have limited lifespans, quickly losing their phenotype and hormone responsiveness. In contrast, organoids retain these characteristics and demonstrate significant expansion potential, overcoming the senescence seen in primary cell cultures (13). EEGOs are expected to reveal key intracellular signaling pathways and depict the overall morphological features of tissues, thereby significantly enhancing our understanding of human endometrial physiology and pathology at the cellular level (14).

Establishing an *in vitro* model for endometrial infection will advance mechanistic studies of endometritis and related infertility factors. While prior research on EEGOs exists, few have applied them to infection studies. Our goal was to develop an infection model using EEGOs that mirror *in vivo* glandular epithelium in expression patterns, structure, hormone responsiveness, and secretion characteristics. We used *E. coli* to induce inflammation and investigated how E2 treatment mitigates post-infection damage. Our results indicate significant anti-inflammatory effects of estrogen and explore its interaction with endometrial receptivity, potentially informing adjunctive therapies for uterine-related diseases. This study underscores the robustness and physiological relevance of our organoid models, particularly during the secretory phase, for studying inflammation in this infection model.

## 2 Materials and methods

### 2.1 Establishment of EEGOs

This study was conducted in accordance with the Declaration of Helsinki. All analyses involving patient and human samples followed the norms and procedures of the Beijing Obstetrics and Gynecology Hospital, Capital Medical University. The appropriate licenses and protocols were approved by the Institutional Review Board (IRB number: 2023-KY-011-02), and informed consent was obtained from the patients involved. Endometrial samples were collected from premenopausal women (*n* = 3) undergoing routine hysterectomy for benign uterine conditions. They had not taken any hormonal drugs in the 3 months preceding the tissue harvest. Endometrial samples were histologically classified as normal proliferative by pathologists at Beijing Obstetrics and Gynecology Hospital. Fresh endometrial tissue was collected during surgeries and processed immediately. The tissues were enzymatically digested at 37°C for 45 min using a combination of collagenase II (1 mg/ml, Sigma, C2-28-100MG) and collagenase IV (1 mg/ml, Sigma, C4-28-100MG), along with a 10 μM ROCK inhibitor (Y27632, MCE, HY-10071/CS-0131), followed by filtration to remove undigested material. The cells were centrifuged at 1,200 rpm following digestion. The final pellet was resuspended in Matrigel to make the final concentration 70% Matrigel (Corning, 356,255, no phenol red, protein concentration was10.5 mg/ml), 3 drops (each drop is about 15 ul) were plated in each well of 24-well plate. The Matrigel was polymerized at 37°C for 30 min before the addition of culture medium to ensure proper matrix formation and overlayed with 500 μl of media containing DMEM/F12(no phenol red, HyClone,SH30272.01), B27(1X, Gibco,17504-044), N2 supplement(1X, Gibco, 17502-048), 500 nM A83-01 (MCE, HY-10432), 250 μg/ml EGF(MCE, HY-P7109), 250 μg/ml R-Spondin-1 (MCE, HY-P7114), 100 μg/ml Noggin (MCE, HY-P7051A). Organoids formed within 3–4 days and were passaged based on growth and confluence, optical microscope, fluorescence microscope (OLYMPUS).

### 2.2 Hormone treatment of organoids

To examine the hormone responsiveness of EEGOs following passaging, 10,000 cells per Matrigel droplet were plated in 24-well plates (3 droplets per well) and allowed to establish into organoids over 3 days in organoid medium. We chose 10 nM E2 (estradiol) and the corresponding concentration of P4 (progesterone) based on previous studies that demonstrated these levels effectively simulate the hormonal environment necessary for the physiological functions of endometrial cells *in vitro* (12). EEGOs were then treated with either 10 nM estradiol (MCE, HY-B0141) or control for 3 days. Following this, organoids were treated with either 10 nM E2 and 1 μM P4 (Progesterone, MCE, HY-N0437) (E2 + P4), 10 nM E2, for a further 4 days. Each treatment was performed in triplicate wells with a fourth well for each treatment used for histology purposes.

### 2.3 Establishment of air-liquid interface (ALI) culture system

Organoids were removed from Matrigel and trypsinized with TrypLE (Gibco, 12604021). The dissociation was performed at 37°C for 5–10 min. Cells were diluted in trypan blue to exclude dead cells and counted using a haemocytometer. A total of 5 × 10⁵ cells were seeded onto membranes of 12-well transwell inserts (0.4 μm, Thermo, 141002) and overlaid with 250 μl of medium. To ensure consistency across experiments, the medium used for the ALI culture is identical to the organoid culture medium. Then, an additional 500 μl of medium was added to the lower chamber. For ALI culture, the medium in the apical compartment was removed 72 h after seeding, and the culture was further maintained with 500 μl of medium in the basolateral compartment and air in the apical compartment. The medium was changed every 3 days.

### 2.4 Co-culture of organoids with bacteria

*E. coli (*ATCC-25922) was maintained by the Microecology Laboratory, Beijing Obstetrics and Gynecology Hospital, and was routinely grown on LB agar at 37◦C. Isolated colonies were picked, inoculated in LB (Solarbio, L8291), and EEGOs were released from the Matrigel using the cell recovery solution, washed with DMEM/F12, and then resuspended in the organoid medium. The *E. coli* concentration was measured using a spectrophotometer (OD600). The bacteria were used in their exponential phase to ensure maximum viability and infectivity. We used an MOI of 1 for the infection experiments. Subsequently, EEGOs were mixed with bacterial cells by rotating (Servicebio, SYC-FZ100) for 1 h in a tissue culture incubator. Following incubation, excess bacterial cells were removed by thorough washing with PBS. The organoids were allowed to recover for 24 h, the medium was replenished every other day. At the designated timepoints, 500 μl of culture medium supernatant was harvested for detection of ELISA. All experiments were repeated three times.

### 2.5 Immunohistochemistry and immunofluorescence

EEGOs were extracted using cell recovery solution (Corning, 354270) for 60 min to preserve integrity and enhance antibody penetration. The organoids were centrifuged at 1,200 rpm for 5 min between washing steps to pellet the organoids and remove any supernatant.Following extraction, organoids were fixed with 4% paraformaldehyde at 4°C for 45 min, rinsed briefly in 1  ×  PBS, stored in 70% EtOH and processed for standard paraffin embedding and sectioning at 5 μm. Paraffin sections were then processed for hematoxylin and EEGOsin (H&E), periodic acid-Schiff (PAS, Beyotime, C0142S) stains and immunohistochemistry. The primary cells are cultured on glass coverslips with 4% paraformaldehyde for 10–15 min at room temperature, followed by three washes with PBS. Permeabilization is done with 0.1%–0.5% Triton X-100 for 5–10 min at room temperature, and cells are again washed with PBS. Blocking is performed using 5%–10% serum for 1 h at room temperature to reduce non-specific binding. The primary antibodies used were estrogen receptors (ER; 1:100, HY-P80663, MCE), progesterone receptors (PR; 1:100, HY-P82121, MCE), Ki67 (1:500, HY-P80506, MCE), MUC1 (1:400, ab4516, Abcam), Pan-Keratin (C11) (1:100, 4545, CST), Vimentin (CST, 5741), E-cadherin (1:250, 3195, CST). EpCAM (1:500, ab223582, Abcam). The secondary antibodies used were Alexa Fluor 555-labeled Donkey Anti-Mouse IgG (H + L) (1:500, A0460, Beyotime) and Alexa Fluor 488-labeled Goat Anti-Mouse IgG (H + L) (1:200, A0428, Beyotime). Nuclei were counter stained with 4′, 6-diamidino-2-phenylindole (DAPI; 10 μg/ml, C1002, Beyotime) for 15 min.

### 2.6 Total RNA isolation and real-time fluorescence quantitative PCR

Total RNA was extracted from the organoids using TRIzol reagent (RNA Extraction Kit, AG11701, Accurate Biology Co., Ltd.). RNA concentration and purity were measured using a NanoDrop spectrophotometer (Thermo Fisher Scientific, ND-2000), ensuring an A260/A280 ratio of 1.8–2.0. Reverse transcription was performed using the appropriate kit (AG11728, Accurate Biology Co., Ltd.), following the manufacturer's protocol. cDNA was synthesized from 500 ng of total RNA in a reaction volume of 20 μl. Real-time quantitative PCR (qPCR) was conducted with SYBR Green (AG11701, Accurate Biology Co., Ltd.) on the ABI7500 system. The cycling conditions were as follows: 95°C for 10 min, followed by 40 cycles of 95°C for 5 s and 60°C for 30 s. Relative gene expression levels were calculated using the 2^-ΔΔCt method, with GAPDH serving as the endogenous control. The primers used for qPCR are listed in Table 1. To ensure sufficient RNA concentration, multiple wells were pooled per group, with each well containing three drops of organoids. Organoids were collected 1-h post-infection with *E. coli.* Separation of *E. coli* from organoids was achieved by differential centrifugation and washing with PBS. The organoids were then pelleted and lysed using TRIzol for RNA extraction. For each qPCR reaction, 1 µg of cDNA was used, with each assay conducted in triplicate. The experiment was repeated three times to confirm the consistency of the results.

**Table 1:** Primers used for qPCR.

Primer	Accession number	Product size	Sequence (5′-3′)
Human_ ESR	XM_054354491	147	F: TGCCCTACTACCTGGAGAAC
			R: CCATAGCCATACTTCCCTTGTC
Human_ PGR	XM_054369129	106	F: GAAGGGCAGCACAACTACTTA
			R: CAGCCTGACAGCACTTTCTA
Human_ ITGβ1	NM_033668	235	F: TGTCCCACTGGTCCAGACA
			R: GACCAGCTTTACGTCCGTAGTT
Human_ LIF	NM_002309	233	F: TCTTGGCGGCAGGAGTTG
			R: CTTGTCCAGGTTGTTGGGGA
Human_ PAEP	NM_001018049	296	F: GCGACCAACAACATCTCCCT
			R: GCCAGGTACTGGCACATCAT
Human_MKI67	NM_001145966	736	F: CTGACCCTGATGAGAGTGAGGGA
			R: TCTCCCCTTTTGAGAGGCGT
Human_MUC1	NM_001371720	119	F: TGCTTACAGCTACCACAGCC
			R: GCTGGGCACTGAACTTCTCT
Human_HSPA1B	NM_005346	131	F: GCGAGGCGGACAAGAAGAA
			R: GATGGGGTTACACACCTGCT
Human_GADD45A	NM_001924	145	F: GAGAGCAGAAGACCGAAAGGA
			R: CACAACACCACGTTATCGGG
Human_DDIT3	NM_001195055	116	F: GGAAACAGAGTGGTCATTCCC
			R: CTGCTTGAGCCGTTCATTCTC
Human_DUSP1	NM_004417	86	F: GCCTTGCTTACCTTATGAGGAC
			R: GGGAGAGATGATGCTTCGCC
Human_ ATF4	NM_182810	153	F: ATGACCGAAATGAGCTTCCTG
			R: GCTGGAGAACCCATGAGGT
Human_ JUNB	NM_002229	128	F: ACAAACTCCTGAAACCGAGCC
			R: CGAGCCCTGACCAGAAAAGTA
Human_ MAPK	NM_001286124	168	F: CGAGCCAGGCAGTGATTTGA
			R: CAGTAACGAGGGAGGGCTTC
Human_IL8	NM_000584	248	F: TCTGCAGCTCTGTGTGAAGG
			R: TTCTCAGCCCTCTTCAAAAACT
Human_ZO1	XM_054378703	218	F: CTCAAGAGGAAGCTGTGGGT
			R: AATCCAGGAGCCCTGTGAAG
Human_ TNF-α	NM_000594	185	F: CACAGTGAAGTGCTGGCAAC
			R: AGGAAGGCCTAAGGTCCACT
Human_IL6	XM_054358146	166	F: CCAGTTGCCTTCTCCCTGG
			R: CTGAGATGCCGTCGAGGATG
Human_IL-1β	NM_000576	125	F: CCAAACCTCTTCGAGGCACA
			R: AGCCATCATTTCACTGGCGA
Human_ GAPDH	NM_001357943	110	F: GACACCCACTCCTCCACCTTT
			R: ACCACCCTGTTGCTGTAGCC

Gene annotation: ESR, estrogen receptor; PGR, progesterone receptor; ITGβ1, integrin subunit beta 1; LIF, LIF interleukin 6 family cytokin; PAEP, progestagen associated endometrial protein; MKI67, marker of proliferation Ki-67; MUC1, mucin 1, cell surface associated; HSPA1B, heat shock protein family A (Hsp70) member 1B; GADD45A, growth arrest and DNA damage inducible alpha; DDIT3, DNA, damage inducible transcript 3; DUSP1, dual specificity phosphatase 1; ATF4, activating transcription factor 4; JUNB, JunB proto-oncogene, AP-1 transcription factor subunit; JUND, JunD proto-oncogene; AP-1 transcription factor subunit; MAPK, mitogen-activated protein kinase; IL8, interleukin-8; ZO-1, tight junction protein 1; TNF-α, tumor necrosis factor; IL-6, interleukin-6; IL-1β, interleukin-1β; GAPDH, glyceraldehyde-3-phosphate dehydrogenase.

### 2.7 RNA-seq analysis

Total RNA was extracted from EEGOs using the Trizol RNA reagent. RNA integrity was evaluated with the RNA Nano 6000 Assay Kit on the Bioanalyzer 2100 system (Agilent Technologies, CA, USA). The quality control parameters for this study included an A260/A280 ratio ≥1.8, an A260/A230 ratio ≥2.0, and an RNA integrity number ≥8.0. A cDNA library was constructed, and RNA sequencing was conducted on the Illumina system. The raw data were processed using an internal computational pipeline. Differentially expressed genes were identified based on a fold change >2 and a false discovery rate (FDR) <0.05. KEGG analyses were performed using the clusterProfiler R package, with an FDR threshold set at 0.05.

### 2.8 ELISA assay

The assay utilized antibodies specific for human TNF-α (NeoBioscience, ECC102a), IL-8 (NeoBioscience, EHC008.96), and IFN-γ (NeoBioscience, EHC102 g.96), immobilized in 96-well plates according to the manufacturer's instructions. Organoid-conditioned medium and protein standards were added to the wells, and samples were diluted as necessary. Target proteins in the medium bound to the immobilized antibodies over 2.5 h. Subsequently, biotinylated secondary antibodies were incubated for 1 h, followed by four washes with 1 × wash solution. The optical density was measured at 450 nm using a microplate reader (TECAN, Profiblot48). Standard curves were generated to quantify cytokine concentrations in the samples. All experiments were repeated three times.

### 2.9 Scanning electron microscopy and transmission electron microscopy

For scanning electron microscopy (SEM), organoids are first fixed in 2.5% glutaraldehyde at least 24 h at 4°C, followed by three washes in phosphate buffer. Post-fixation is carried out in 1% osmium tetroxide for 1 h, with subsequent washing. The organoids are then dehydrated through a graded ethanol series (30%–100%), critically point dried, and coated with a thin layer of gold or platinum using a sputter coater. Finally, the samples are imaged using SEM to examine surface morphology. For transmission electron microscopy (TEM), organoids undergo a similar fixation and post-fixation process. After osmium tetroxide treatment, Tissues were then dehydrated and embedded, and then cut into 70-nm thick slides, which were embedded in PON812 epoxy resin (SPI, West Chester, PA, USA) and observed under transmission electron microscopy (JEM1230, JEOL Co., Hitachi Ltd., Tokyo, Japan). The ultrastructure morphological features within the epithelium, such as endoplasmic reticula and mitochondria, were observed and photographed independently by 2 investigators.

### 2.10 Statistical analysis

Comparisons between groups were determined with the two-sided unpaired Student's *t*-test. Significant differences among the three sets of experiments were determined using one-way analysis of variance (ANOVA). All statistical analyses were conducted using GraphPad Prism 9, and the data are presented as mean ± SEM. Differences were considered significant at *P* < 0.05.

## 3 Results

### 3.1 Organoid construction for long-term stable passaging

**Figure 1:**
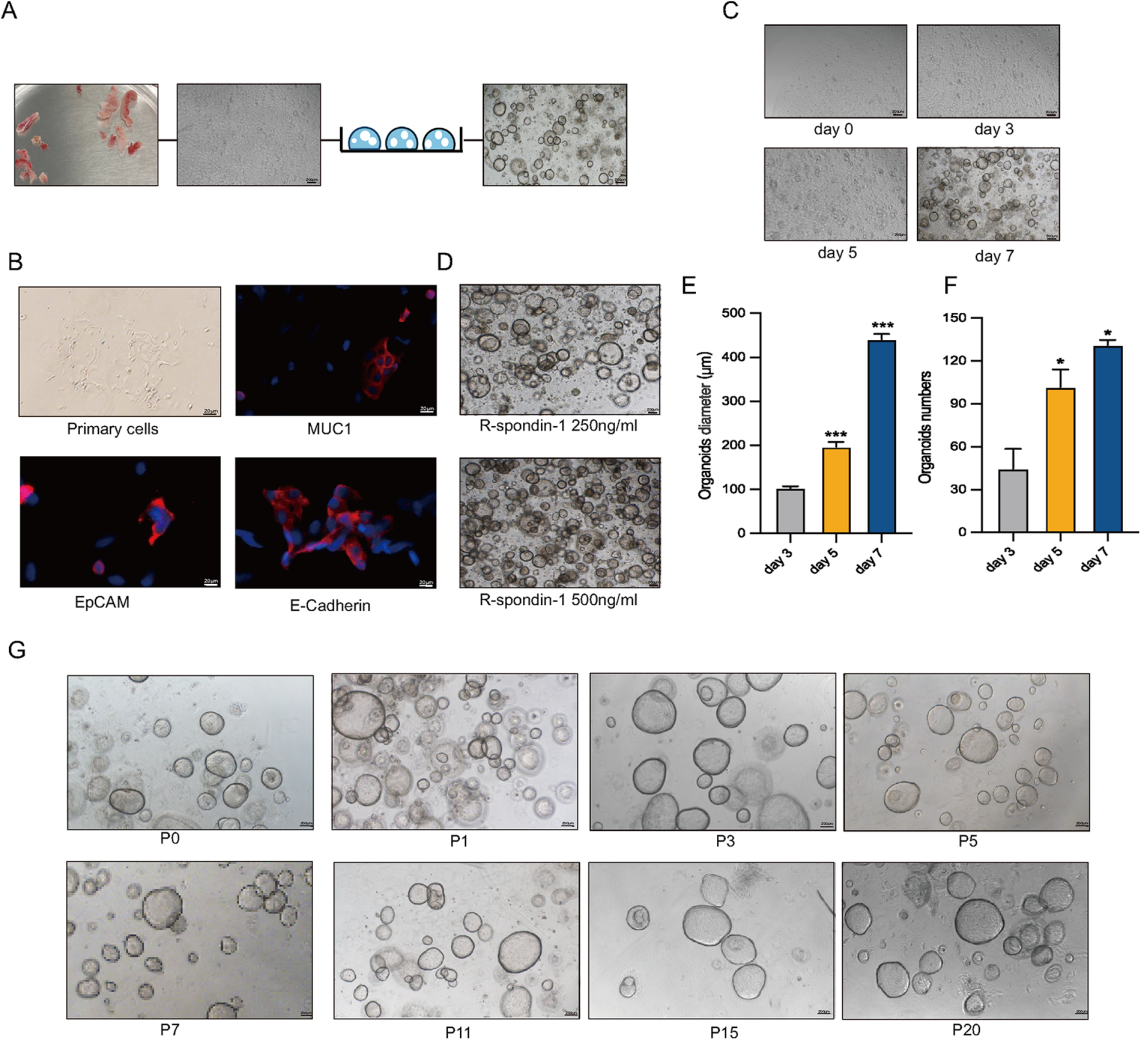
Long-term stable passage of organoids. **(A)** Workflow for the isolation of human endometrial epithelial glands and culture in a 3D extracellular Matrigel plug. Collect human endometrial tissue, obtain human endometrial primary cells, and construct EEGOs. Scale bar: 100 μm. **(B)** Immunofluorescence microscopy of MUC1 (red), E-Cadherin (red), EpCAM (red), and DNA (blue) in human primary endometrial cells. Scale bar: 20 μm. **(C)** Brightfield images of organoids formed within 8 days. Scale bar: 200 μm. **(D)** Brightfield images of organoids under 500 ng/ml and 250 ng/ml R-spondin conditions. Scale bar: 200 μm. **(E)** Diameter of organoids on days 3, 5, and 7. **(F)** Number of organoids per drop of Matrigel on days 3, 5, and 7. **(G)** Brightfield images of different passages demonstrating the long-term expansion of EEGOs. Scale bar: 200 μm. The results are shown as the mean ± SEMs of triplicate samples and are representative of three independent experiments. *P ≤ *0.05*, **P ≤ *0.01*, ***P ≤ *0.001*.

Human endometrial epithelial glands were isolated and cultured in a three-dimensional Matrigel with conditioned medium (Figure 1A). Immunofluorescence staining revealed strong expression of MUC1, E-Cadherin, and EpCAM in epithelial cells and gland fragments isolated from endometrial biopsies (Figure 1B). In a specific culture system utilizing Matrigel and conditions, EEGOs were generated from endometrial tissues and readily formed within a week, growing into large spherical structures (Figure 1C). Given that R-Spondin-1 amplifies the Wnt/β-catenin signaling pathway, maintaining stemness while promoting the self-renewal of stem cells, R-Spondin-1 concentrations of 500 ng/ml and 250 ng/ml were selected for culturing the organoids. While the higher concentration allows for more organoids, the limited space in the Matrigel impedes further expansion of the organoids. Therefore, the organoids cultured with 250 ng/ml R-Spondin-1 grew better (Figure 1D). Organoid counts and diameter measurements were conducted on days 3, 5, and 7 respectively (Figures 1E,F). Organoids on day 5 exhibited statistically significant differences in number and growth diameter compared to day 3 (*p* < 0.05). After 8–10 days of culture, the organoids expanded at a 1:2 or 1:4 ratio, then dissociated into cell clusters that regenerated into new organoids. After 1–2 passages, no stromal cells were observed, and the organoids have now exceeded 20 passages, maintaining a rounded cystic structure (Figure 1G).

### 3.2 Human EEGOs recapitulate the *in vivo* phenotype of endometrial glands

**Figure 2:**
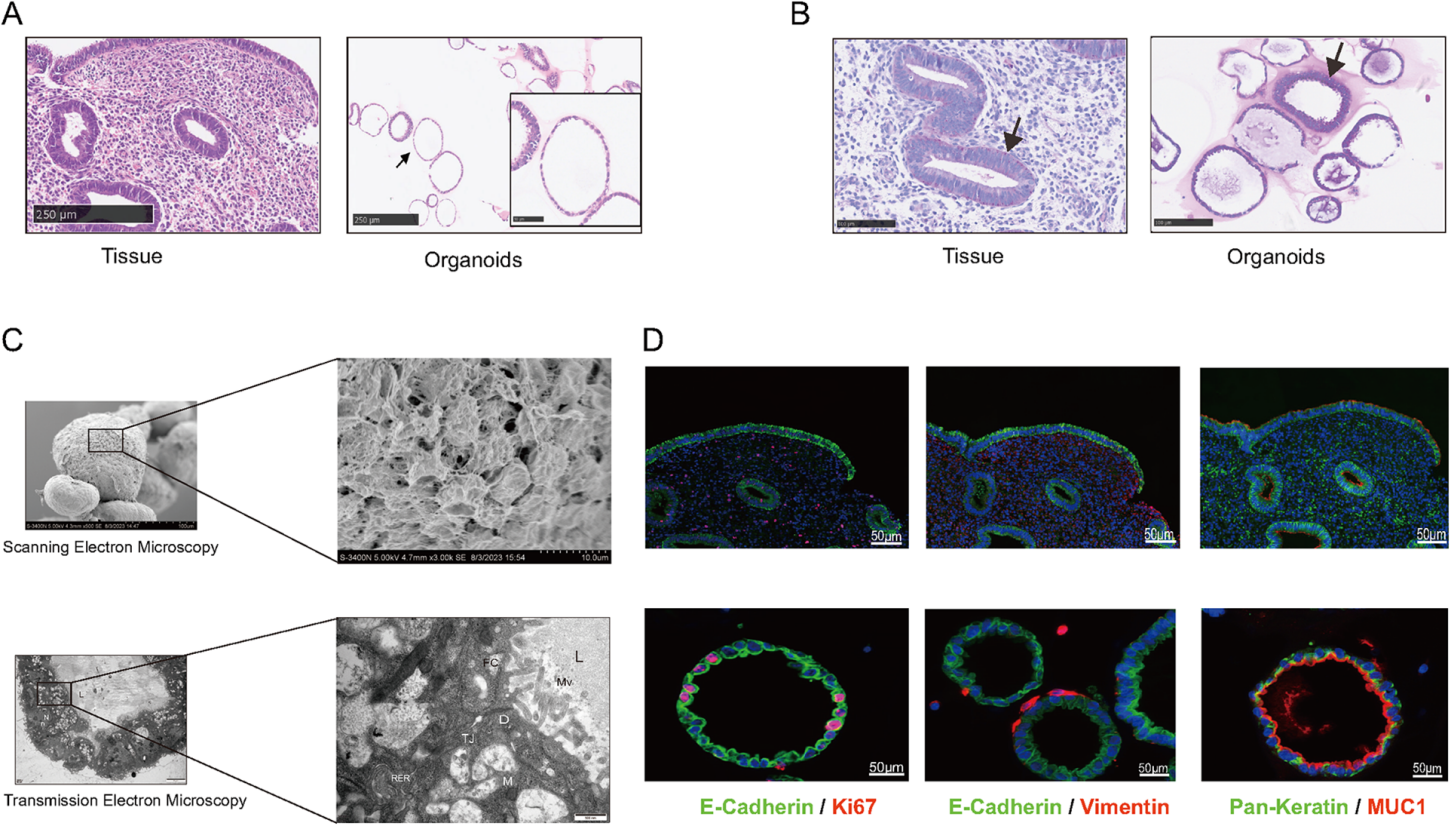
Human EEGOs recapitulate the *in vivo* phenotype of endometrial glands. **(A,B)** Histological [hematoxylin and eosin in (H&E)] analysis, EEGOs show a single-layered columnar epithelium. Arrows point to EEGO. Immunohistochemical examination of endometrial markers, and mucin detection (PAS staining) in endometrial tissue and EEGOs. Immunohistochemistry analysis of laminin in organoids. Arrows point to mucus production (PAS). **(C)** A Scanning Electron Microscope (SEM) image revealing that organoids are spherical cystic structures with tight cell junctions. The boxed areas are magnified as indicated. Scale bars: 10 μm. A Transmission Electron Microscope (TEM) image revealing glandular-like morphology and the presence of microvilli and cilia in EEGOs. The boxed areas are magnified as indicated. Scale bars, 500 nm. Ultrastructure of epithelial glandular organoids recapitulating the physiological architecture of glands in human healthy endometrium. L, lumen; N, nucleus; M, mitochondria; Mv, microvilli; TJ, tight junction; D, desmosomes; n, nucleolus; RER, rough endoplasmic reticulum; fc, filaments of cytoskeleton. **(D)** EEGOs were established from the human endometrial epithelium. The expression of the epithelial cell marker MUC1 and E-cadherin was determined by immunofluorescence, proliferation marker Ki67 was also detected, and stromal marker vimentin was detected. Round organoids formed under specific culture conditions as described in Materials and Methods and express MUC1, E-cadherin (CDH1), and Ki67. The developed organoids exhibited morphology and expression of markers resembling human endometrial glands. Tissue scale bar: 100 μm; organoids scale bar: 50 μm.

To *evaluate* the structure of EEGOs and confirm their glandular origin, we assessed the expression of endometrial characteristic markers in the organoids. H&E staining of the organoids confirmed the presence of columnar epithelial cells arranged in a monolayer, consistent with the histological features of epithelial tissue (Figure 2A). Periodic acid-Schiff (PAS) staining revealed the presence of glycogen, the primary component of endometrial glandular secretions, highlighting its significance as observed *in vivo* (Figure 2B). Scanning electron microscopy reveals the spherical appearance of the organoid with dense connections between surface cells, while transmission electron microscopy shows a cystic structure lined by columnar epithelium, with visible secretions in the lumen. Microvilli are observed on the pseudocolumnar epithelium, supported by an amorphous basement membrane, with nuclei located at the base. The cytoplasm contains endoplasmic reticulum, Golgi apparatus, and numerous secretory vesicles, with visible secretory activity at the tip, features also found in human endometrial epithelial cells (Figure 2C). Markers of the endometrial epithelium (MUC1, E-Cadherin) were strongly expressed in EEGOs, similar to epithelial cells of the human endometrium. The expression of pan-keratin and vimentin were evaluated to demonstrate the enrichment of epithelial cells in the stroma during organoid development. Pan-Keratin is localized in the cytoplasmic compartment of organoid cells, while vimentin is expressed minimally outside the organoids. Ki67, which is associated with proliferation, is also located in the nuclear compartment of organoid cells (Figure 2D). Consequently, EEGOs closely resemble endometrial glands *in vivo*, confirming their epithelial origin under all experimental conditions.

### 3.3 Characteristic response of EEGOs to hormones

**Figure 3:**
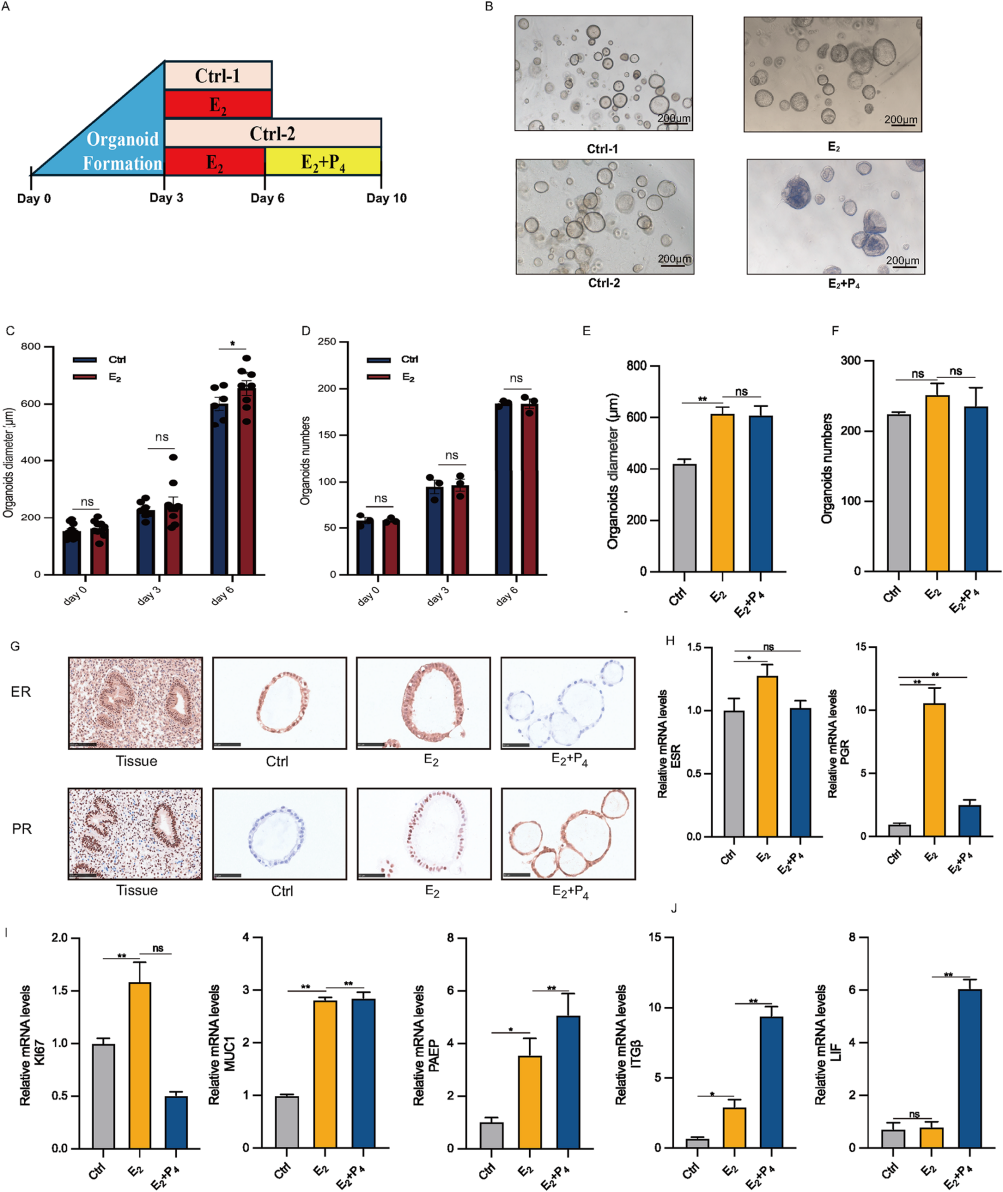
Characteristic responses of EEGO to hormones. **(A)** Organoids were treated with either nothing (Control) or 10 nM E2, followed by either nothing (control), or 10 nM E2 + 1 μM P4. **(B)** EEGOs were treated with hormones mimicking the environment of the proliferative phase (E2), secretory phase (E2 + P4). Representative bright-field images are shown after hormone treatments. Scale bar: 200 μm. **(C)** Diameter of organoids on days 0, 3, and 6 under control and E2 conditions. **(D)** Number of organoids on days 0, 3, and 6 under control and E2 conditions. **(E)** Changes in the diameter of organoids after treatment with 10 nM E2 and 10 nM E2 + 1μM P4. **(F)** Changes in the number of organoids after treatment with 10 nM E2 and 10 nM E2 + 1μM P4. **(G)** Organoids were observed after sex hormone treatment mimicking the follicular phase of a normal menstrual cycle. Levels of ER and PR were detected by immunohistochemical staining in EEGOs. **(H–J)** RT-PCR of RNA isolated from organoid cultures treated as described in methods. Human endometrial epithelial organoids are responsive to ovarian steroid hormones. Relative ESR, PGR, KI67, MUC1, PAEP, ITGβ and LIF mRNA levels in hormone-treated organoids. The results shown are one of three independent experiments. Data are means ± SEMs. ^*^P ≤ *0.05*, **P ≤ *0.01*, ***P ≤ *0.001*.

The most distinctive feature of EEGO is their hormonal responsiveness. To study this *in vitro*, EEGOs were treated with either 10 nM E2 or 10 nM E2 + 1 μM P4 (Figure 3A). Three days post-derivation, treatment with hormones mimicking the proliferative phase (E2) and the secretory phase (E2 + P4) was initiated. No significant morphological differences were observed between the control group and the E2-treated group. However, the E2 + P4 group exhibited significant morphological changes, with the EEGOs curling and folding inward into the lumen and thickening of the luminal wall. This thickening resembled the endometrial changes seen during the secretory phase (Figure 3B). Considering that estrogen can promote the proliferation of EEGOs, we measured the diameters of EEGOs in the control and E2 groups over different days. The difference in the diameters of the EEGOs were statistically significant with the increased duration of E2 treatment (*P* < 0.05). Additionally, we counted the number of EEGOs during the treatment period. There was no statistically significant difference in the number of EEGOs between the two groups, indicating that E2 treatment promotes the proliferation and enlargement of EEGOs without affecting their number (Figures 3C,D). At the end of the E2 and E2 + P4 treatments, we counted the number and measured the diameter of the organoids in the control, E2, and E2 + P4 groups. There was no significant difference in the number of organoids among the three groups. Interestingly, the diameter of the organoids was largest after E2 treatment (*p* < 0.05), and the diameter showed no significant change after E2 + P4 treatment, consistent with the *in vivo* changes of endometrial epithelial cells during the proliferative and secretory phases (Figures 3E,F). IHC staining of the organoids was conducted to examine the expression of ER and PR. Treatment with E2 increased the number of ER and PR positive cells compared to the untreated control organoids. In contrast, the number of ER positive cells was significantly lower in the E2 + P4 treated organoids. The reduction and absence of ER in the E2 + P4 treated organoids were consistent with the loss of ER observed in the endometrium between the proliferative and secretory phases of the menstrual cycle (Figure 3G). Next, we used real-time quantitative PCR to measure established E2 and P4 stimulated genes. E2 treatment increased ESR and PGR mRNA levels in the organoids, while a decrease in these gene expressions was observed in the E2 + P4 treatment (Figure 3H). The Ki67 proliferation markers showed that the mRNA expression of Ki67 increased with E2 treatment compared to the control organoids, while treatment with E2 + P4 decreased it. This was consistent with observations in the endometrium between the proliferative and secretory phases of the menstrual cycle. For the glandular marker MUC1, E2 and E2 + P4 treatments resulted in increased mRNA expression, with no significant difference between the two treatments (Figure 3I). For P4-responsive genes, PAEP, LIF, and ITG*β* mRNA levels were significantly increased in the E2 + P4 treated organoids. A slight increase in these genes was observed in the organoids treated with E2 (Figure 3J).

### 3.4 Establishment of EEGO infection models

**Figure 4:**
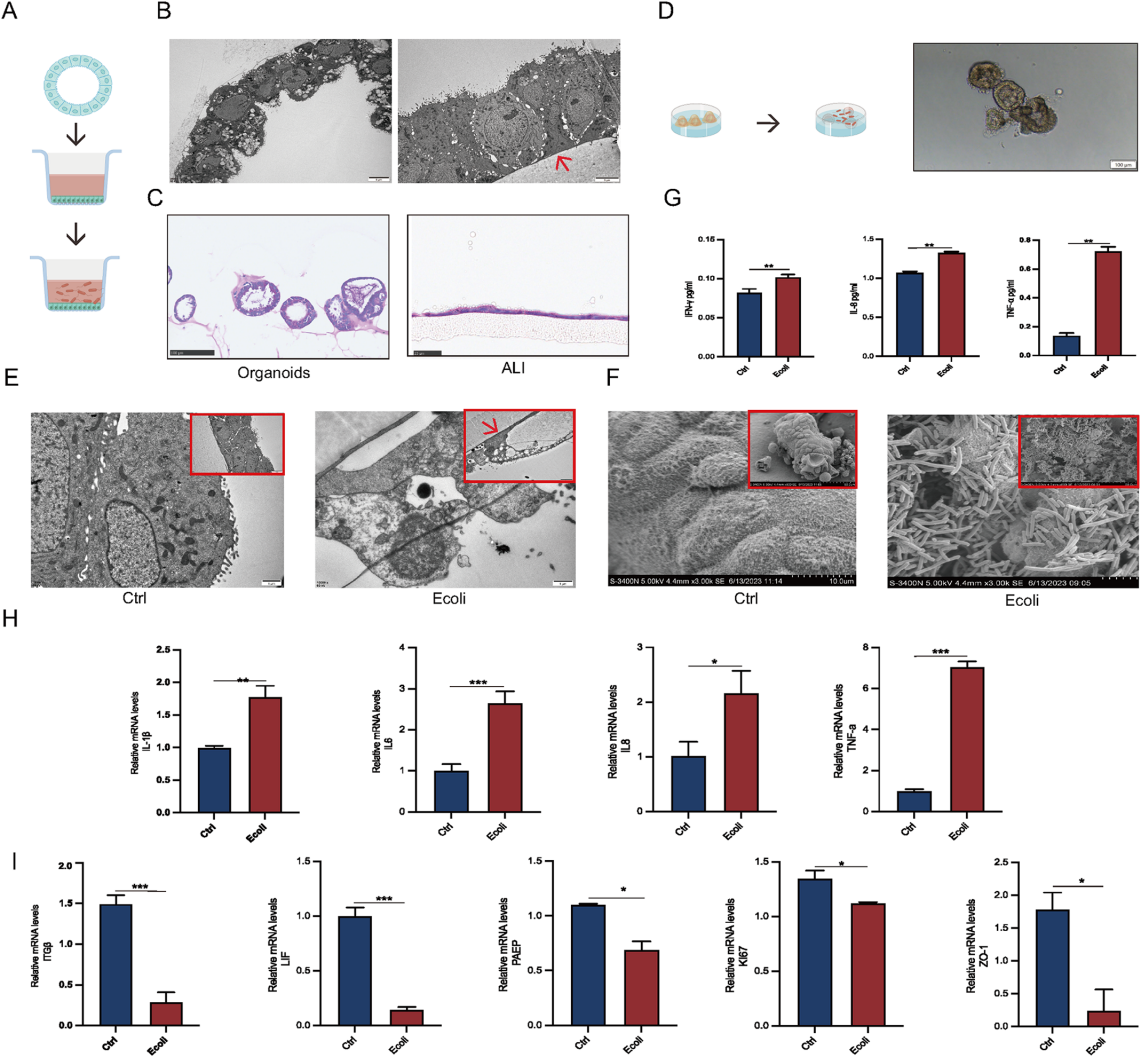
Establishment of EEGO infection models. **(A)** Workflow for air-liquid interface (ALI) culture of EEGOs. **(B)** Scanning electron microscopy images comparing EEGOs before and after ALI culture. After ALI culture, the previously hollow, cystic structure of the EEGOs expanded into a single-layered columnar epithelium with nuclei located at the basal side. The red arrow indicates the ALI chamber membrane. Scale bar: 5 μm. **(C)** Hematoxylin and EEGOs in (H&E) staining of EEGOs show that after ALI culture, the organoids transformed from a spherical structure into a single-layered columnar epithelium. Organoid scale bar: 100 μm; ALI scale bar: 25 μm. **(D)** Workflow for direct infection of EEGOs with *E. coli.* Scale bar: 100 μm. **(E)** EEGOs after ALI culture and *E. coli* infection (Control vs. *E. coli*). Organoids show cell fragmentation and loss of intact morphology. The red arrow indicates the ALI chamber membrane. Scale bar: 1 μm. **(F)** EEGOs directly infected with *E. coli*. Scale bar: 10 μm. **(G)** ELISA assay of inflammatory factor expression. **(H)** RT-qPCR assay of mRNA expression of inflammatory factors. **(I)** RT-qPCR assay of mRNA expression of P4 hormone response genes, proliferation-related genes, and tight junction-related genes in organoids. The results shown are one of three independent experiments. Data are means ± SEMs. ^*^P ≤ *0.05*, **P ≤ *0.01*, ***P ≤ *0.001*.

To better simulate the infection process of human endometrial epithelium, EEGOs, with their epithelial surface positioned within the lumen, were subjected to air-liquid interface (ALI) culture. This involved releasing the organoids from the Matrigel and placing them into ALI chambers (Figure 4A). Projection electron microscopy indicated that while the epithelial surface in EEGOs is oriented towards the lumen, after ALI culture, the epithelial surface turned outward, which is more conducive to *E. coli* adhesion (Figure 4B). H&E staining was performed before and after organoid treatment to observe morphological changes. Organoids are typically hollow spherical structures. However, when cultured using an ALI, a monolayer of cells is covered with a chamber membrane (Figure 4C). Direct infection of the organoids showed that *E. coli* surrounded the organoids, leading to darker color and disrupted luminal wall integrity compared to controls (Figure 4D). Post-infection TEM revealed disrupted cell morphology in the experimental group, with loss of intercellular tight junctions and fragmented cell nuclei, compared to the control group (Figure 4E). Scanning electron microscopy confirmed that *E. coli* adhered to the surface of the organoids, causing surface cell wrinkling (Figure 4F). Based on the results mentioned earlier, the number of cells cultured using the ALI system is lower compared to organoids, and nearly all cells die following infection. Therefore, direct infection is employed for examination throughout the manuscript. Post-infection, cell supernatants were collected for ELISA assays, showing significantly elevated expression of TNF-α, IL-8, and IFN-γ compared to controls (*p* < 0.05) (Figure 4G). Additionally, mRNA expression levels of inflammatory factors (IL-1β, IL-6, IL-8, TNF-α) were significantly upregulated (*p* < 0.05) (Figure 4H). qPCR results confirmed that acute infection disrupts endometrial receptivity (PAEP, LIF, ITGβ), affects cell proliferation (Ki67), and disrupts intercellular tight junctions (ZO-1) (*p* < 0.05) (Figure 4I).

### 3.5 Signaling pathway alterations after infection of EEGO

**Figure 5:**
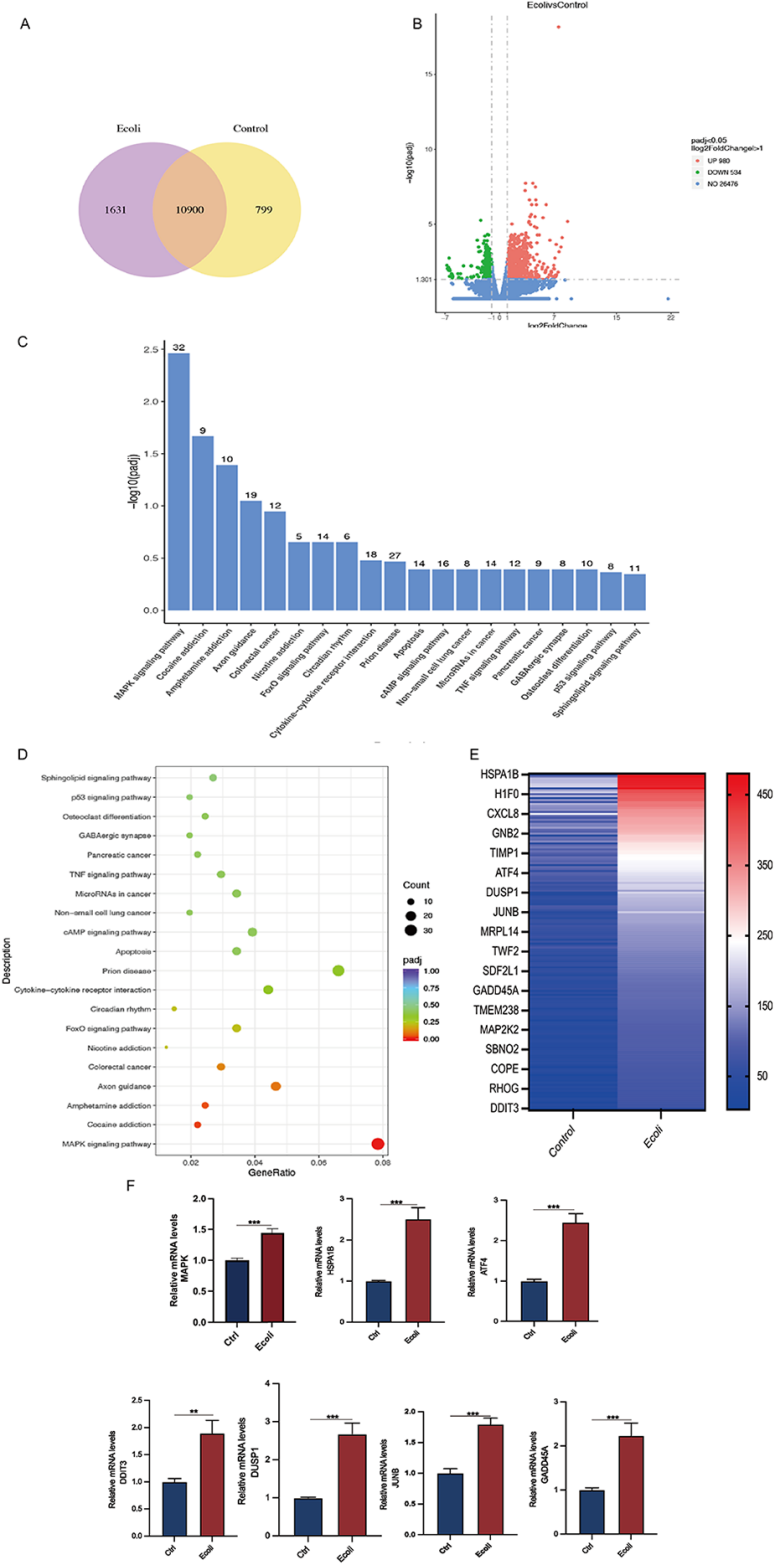
Differential gene expression and pathway analysis in organoids infected with *E. coli.* **(A)** Venn diagram of co-expressed genes per comparison (*E. coli* vs. Control). The selection criteria were *p* < 0.05 and fold change contrast ≥ 2. **(B)** Volcano plots of differentially expressed genes, with 980 genes upregulated and 534 genes downregulated (*E. coli* vs. Control). **(C)** KEGG analysis of differentially expressed mRNA target genes. A bar chart of the top 20 KEGG pathways with upregulated genes is presented. KEGG: Kyoto Encyclopedia of Genes and Genomes. **(D)** Dot plot representing key signaling pathway changes in organoids post-infection. Dot size indicates the proportion of cells in the cluster expressing a gene, and shading indicates the relative level of expression (light to dark). **(E)** Hierarchical clustering comparing *E. coli* vs. Control of significantly regulated genes (2-fold, FDR *P* < 0.05). **(F)** RT-PCR of RNA isolated from organoid cultures treated as described in methods. Relative MAPK, HAPA1B, ATF4, DDIT3, DUSP1, JUNB, and GADD45A mRNA levels in *E. coli*-treated organoids. The results are shown as the mean ± SEMs of triplicate samples and are representative of three independent experiments. *P ≤ *0.05*, **P ≤ *0.01*, ***P ≤ *0.001*.

High-throughput mRNA sequencing (mRNA-seq) of the untreated and *E. coli*-treated organoids revealed 10,900 co-expressed genes between the two groups, as shown in a Venn diagram (Figure 5A). Using a threshold of 1.5-fold change and a false discovery rate of <0.05, compared to the control group, 980 genes were upregulated, and 534 genes were downregulated in the *E. coli*-treated group (Figure 5B). The top 20 significantly upregulated KEGG pathways were selected from the enrichment results of the infected group, and a bar chart was created. Analysis revealed predominant enrichment of the MAPK signaling pathway post-infection in the KEGG pathway analysis. (Figures 5C,D). Upregulated genes among the differentially expressed genes were selected to create a heat map and validated using qPCR. Post-infection, members of the heat shock protein 70 (HSP70) family, specifically HSPA1B, and transcription factors ATF4, DDIT3, and JUNB showed increased expression (*p* < 0.05). Additionally, the expression of GADD45A, a gene related to DNA damage repair, was also elevated (*p* < 0.05) (Figures 5E,F).

### 3.6 The effect of estrogen on post-infection EEGO

**Figure 6:**
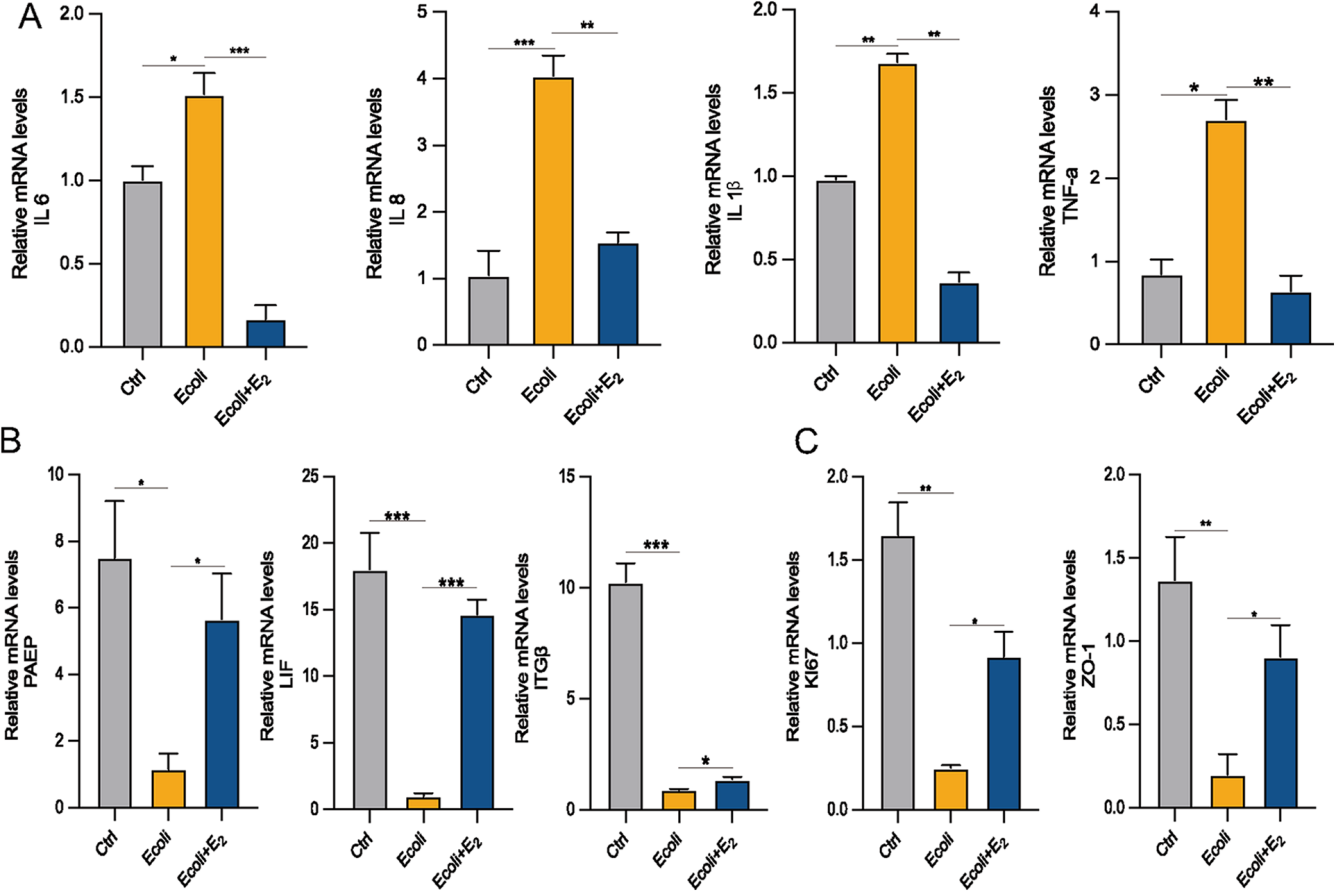
Effect of E2 on *E. coli*-induced inflammation in EEGOs. **(A)** RT-qPCR assay of mRNA expression of inflammatory factors. **(B)** RT-qPCR assay of mRNA expression of P4 hormone response genes. **(C)** Expression of proliferation- and tight junction-related genes in organoids. The results are shown as the mean ± SD and are representative of three independent experiments. ^*^P ≤ *0.05*, **P ≤ *0.01*, ***P ≤ *0.001*.

Estrogen exerts anti-inflammatory effects by reducing the production of pro-inflammatory cytokines (15). To determine whether E2 could alleviate inflammation, *E. coli*-infected organoids were incubated and cultured for 24 h with the addition of E2. The mRNA expression of inflammation-associated factors IL-6, IL-8 (which is CXCL8 in Figure 5E), IL-1β and TNF-α increased in the *E. coli*-exposed group compared to the control group (*P* < *0.05*). However, the mRNA expression of IL-1β and IL-6 were significantly lower in the *E. coli* + E2 group compared to the *E. coli* group without E2 treatment (*P* < 0.05). These findings suggest that E2 attenuates inflammatory damage to the endometrium caused by bacterial infection (Figure 6A). Further exploring the effect of estrogen on the recovery of receptivity, we found that the receptivity of infected organoids improved significantly after estrogen treatment. Specifically, the mRNA expression levels of PAEP, LIF, and ITG*β* were significantly elevated in the *E. coli* + E2 group compared to the *E. coli* group without estrogen treatment (*P* < 0.05), further supporting the role of estrogen in enhancing endometrial receptivity (Figure 6B). To investigate the proliferation and barrier repair capacity of organoids after inflammatory injury, the expression of the cellular tight junction-related gene ZO-1 was significantly up-regulated after E2 treatment compared to the *E. coli*-exposed group (*P* < 0.05). Additionally, the expression of the proliferation-related gene Ki67 was significantly increased (*P* < 0.05). These findings suggest that organoids have innate advantages in proliferation and barrier repair, making them promising tools for *in vitro* studies (Figure 6C).

## 4 Discussion

The endometrium serves as the site for embryo implantation and development. Traditionally, the uterine cavity was considered a sterile environment distinct from the vagina. Modern views suggest that the endometrium harbors hundreds of microbial species, dynamically regulating its microbiota to ensure the formation of a maternal-fetal interface microenvironment (16–18). In addition to microbial disruptions of endometrial homeostasis and metabolic functions, inflammation is a significant contributing factor. Endometritis manifests as acute or chronic forms. Acute endometritis typically involves sudden onset symptoms like fever, lower abdominal pain, and abnormal vaginal discharge, often following uterine procedures, childbirth, or miscarriage. Untreated acute cases can progress to chronic endometritis, which may present with mild, nonspecific symptoms such as increased vaginal discharge or pelvic discomfort, sometimes overlooked in clinical practice. Studies consistently link endometritis to higher rates of infertility in affected women (19), recurrent implantation failure (20), significantly increased rates of recurrent pregnancy loss (21), and increased risks of obstetric and neonatal complications (22). Endometritis is typically caused by Gram-negative bacterial strains, such as *E. coli*. In this study, we chose *E. coli* as a representative pathogen for several reasons. First, *E. coli* is one of the most common bacteria associated with uterine infections, particularly endometritis, and pelvic inflammatory disease (PID). Its presence is clinically relevant in the context of bacterial-induced inflammatory responses in the uterus, as it is frequently found in both acute and chronic endometritis cases. *E. coli* infection triggers endometrial inflammation by releasing lipopolysaccharides (LPS) in the infected uterus (23). LPS binds to the transmembrane receptor TLR4, activating the NF-κB pathway and resulting in the release of inflammatory cytokines (24). In addition to directly impacting human preimplantation embryo cleavage rates, blastocyst formation, and pregnancy success, pro-inflammatory cytokines such as IL-1, IL-6, and TNF-α, mediated by bacterial surface LPS, can influence embryo implantation. While these cytokines are essential for normal immune regulation during implantation, an imbalance or overproduction in response to infection may lead to detrimental effects on the implantation process.

Traditional cell culture offers advantages of simplicity, low cost, and minimal resource requirements compared to three-dimensional organoid culture. However, two-dimensional culture lacks the complex internal tissue structure needed to accurately simulate cellular behaviors, differentiation, and functions, particularly in studying specific tissue processes and disease relevance (25). Moreover, the planar nature of two-dimensional culture restricts crucial cell-cell and cell-matrix interactions (26). Commonly used animal models, such as mice, differ significantly from humans in physiology and immune systems, including distinct reproductive cycles. Notably, mice have a shorter estrous cycle compared to the human menstrual cycle, and their uterine anatomy is bicornuate, unlike the human single-chambered uterus. Additionally, the placental invasion and immune responses during infections differ significantly between the two species, which can influence the interpretation of studies related to reproductive health and disease and entail ethical concerns and high maintenance costs. A defining feature of Matrigel-embedded three-dimensional organoid structures is their polarized epithelium with a central lumen. The apical-in/basal-out polarity of organoids presents a challenge when studying epithelial interactions with embryos or infections by microbes or viruses that reside in the uterine lumen (27, 28). To overcome this limitation, an air-liquid interface (ALI) culture system was developed to reverse polarity, based on methods reported for human airway organoids (29, 30). In contrast, the ALI culture model effectively simulates natural air exposure and tissue structures, particularly suited for studying respiratory and epithelial cells (31). Cells in ALI culture can differentiate into specialized cell types, such as ciliated cells in respiratory models, crucial for studying specific tissue functions and responses (32). In our methodology, we infect cells from the basal side due to the reversed polarity in Matrigel-embedded organoids, which complicates the modeling of natural apical infections. While ALI cultures offer the advantage of facilitating apical infections, we opted for the organoid system in this study because of several limitations associated with ALI. These include scalability issues, the time required for cell differentiation, and the mechanical stress exerted on cells during the culture process. Furthermore, ALI cultures have difficulty replicating the complex three-dimensional architecture and cellular diversity of tissues, limiting their ability to accurately simulate intricate tissue interactions. Additional drawbacks of ALI systems include longer culture periods, cell loss, and the inability to visually monitor cell growth and behavior, which further hinder their practical application in long-term studies. In constructing infection models, organoids allow for direct observation of morphological changes and pathogen adhesion. Organoids also demonstrate unique convenience in handling samples post-infection. Moreover, organoids derived from human endometrial tissue retain the genetic and phenotypic characteristics of the original tissue, enabling more precise studies of human-specific inflammatory responses, such as endometritis, and providing crucial insights for developing treatments. They reduce the need for animal experiments, addressing ethical concerns, and offering a more ethically aligned research option. Despite their initial complexity, organoids have lower long-term maintenance costs, presenting significant cost-effectiveness. In this study, we developed a novel organoid culture technique to establish a bacterial-induced endometritis model. Our findings indicate that the organoids originate from the endometrial glandular epithelium and, like the *in vivo* endometrium, possess secretory functions and hormone responsiveness (33). PAEP is expressed and secreted by human endometrial glandular cells during the secretory phase of the menstrual cycle, with its expression stimulated by P4, and is undetected in the proliferative phase of the endometrium (34). LIF is present in the glands of the secretory phase endometrium, a member of the interleukin-6 cytokine family. Decreased LIF in uterine cavity fluid is associated with infertility in comparison to fertile women, and complete absence of LIF is also linked to recurrent miscarriages (35–37). ITGβ expression begins on day 20 of the menstrual cycle, secreted by luminal epithelial cells and expressed on the surface of the endometrium, consistent with the initiation of the “implantation window,” persisting through pregnancy and expressed on the decidua, serving as a marker for establishing endometrial receptivity and the opening of the implantation window (38, 39). After E2 + P4 treatment, the expression levels of P4-responsive genes increased in the organoids. During the organoid culture construction process, the concentration of various factors is crucial for the growth and maintenance of organoid characteristics (12). For example, our experiments found that the growth of organoids was restricted when the concentration of R-spondin-1 was 500 ng/ml. In limited space, an excessive number of organoids is detrimental to their optimal growth, thus careful consideration of factor concentrations during culture is necessary.

The role of estrogen is well-established (40–43). Many studies have shown that estrogen can influence disease inflammation by either activating or inhibiting inflammatory pathways, potentially playing a dual role in inflammation. Compared to other drugs, E2 is safe and effective for treating endometritis. However, the efficacy of E2 in alleviating endometritis depends on concentration and duration; excessive or prolonged use may exacerbate inflammation. This study demonstrates that after *E. coli* treatment, cells are in a stressed state with increased expression levels of heat shock proteins and transcription factors (ATF4, DUSP1, JUNB), activation of the MAPK signaling pathway, and significant upregulation of inflammation-related genes IL-1β, IL-6 and IL8, which is the same molecule that CXCL8 shown in Figure 5E. However, the expression levels of these genes significantly decrease after E2 treatment. This indicates that E2 treatment can alleviate the inflammatory damage caused by *E. coli* in EEGOs, further supporting the anti-inflammatory properties of E2. The Wnt signaling pathway is a key driver for stem cells in various tissues of mammals, essential for the growth and development of organoids to maintain stemness, form spherical structures, and achieve maturation (44–46). Is E2 also associated with the Wnt signaling pathway in restoring cellular receptivity and exerting anti-inflammatory effects? Several studies have shown that E2 promotes cell proliferation and barrier restoration by activating the Wnt pathway (47). Furthermore, there are reports indicating that E2 can also inhibit the Wnt pathway, thereby impeding tumor development (47). Additionally, it has been reported that estrogen deficiency is associated with increased oxidative stress, which can be mitigated by estrogen administration. Oxidative stress is widely recognized for its negative impact on fertility and its significant role in the development of malignancies, neurodegenerative disorders, cardiovascular diseases, and the aging process (48). Whether E2′s anti-inflammatory effects, promotion of cell proliferation, and barrier repair also involve reducing oxidative stress requires further investigation. Some study found that E2 reduces IL-1β and IL-6 expression and inhibits NF-κB pathway activation, indicating anti-inflammatory effects. Upon receiving external stimuli, such as pro-inflammatory signals, the NF-κB proteins that sequester NF-κB in the cytoplasm are phosphorylated and subsequently degraded, allowing NF-κB to translocate to the nucleus and activate target gene transcription. However, additional research is necessary to elucidate the interactions between NF-κB, the Wnt pathway, and oxidative stress pathways and to (49) establish meaningful connections among them. However, it is important to note that, in our sequencing results, many signaling pathways involved in addiction were identified. This might be because these signaling pathways, such as dopamine or opioid signaling, overlap with inflammatory and immune response pathways. These pathways can influence cellular stress, barrier function, and immune activation, which are highly relevant to the pathological processes we are studying in the context of endometrial inflammation.

To our knowledge, no previous studies have reported on a model of bacterial infection-induced inflammatory response in human EEGOs. Our study demonstrates that EEGOs can serve as a valuable system for studying host-pathogen interactions during bacterial infections, elucidating mechanisms of endometritis and its contributions to infertility and endometrial cancer. One limitation of our study is the inability of organoid models to fully replicate immune cell influx and interactions, which are crucial for accurately simulating *in vivo* conditions. While organoids offer a valuable platform for studying endometrial biology, they primarily consist of epithelial and stromal cells, lacking the complex immune microenvironment of natural tissues. This limitation results in the absence of critical immune-mediated responses, such as immune cell recruitment, cytokine signaling, and pathogen clearance—key elements for a comprehensive understanding of endometrial immune dynamics. As a result, our observations of cellular proliferation, regeneration, and inflammatory signaling may not entirely reflect *in vivo* conditions. To address this, we are investigating strategies like co-culturing organoids with immune cell populations or adding immune-modulating factors to create a more physiologically relevant environment. These approaches aim to enhance the translational value of organoid-based studies by more accurately replicating immune-endometrial interactions. Building on the feasibility demonstrated in this study, we are also developing a more advanced culture system for EEGOs. This includes incorporating immune cells, co-culturing multiple bacterial species to simulate the reproductive microbiome, and evaluating whole-genome gene expression changes. These efforts will lead to a deeper understanding of microenvironmental alterations that contribute to infertility or cancer during infections.

## Data Availability

The original contributions presented in the study are included in the article/Supplementary Material, further inquiries can be directed to the corresponding authors.

## References

[1] Liao J Yang S Chen K Chen H Jiang F Zhang W A predictive model for first-trimester pregnancy inception after IVF-ET based on multimodal ultrasound evaluation of endometrial receptivity. BMC Med Imaging. (2022) 22:158. 10.1186/s12880-022-00863-w 36058920 PMC9441094

[2] Aplin JD Ruane PT. Embryo-epithelium interactions during implantation at a glance. J Cell Sci. (2017) 130(1):15–22. 10.1242/jcs.175943 28043966

[3] Franasiak JM Alecsandru D Forman EJ Gemmell LC Goldberg JM Llarena N A review of the pathophysiology of recurrent implantation failure. Fertil Steril. (2021) 116(6):1436–48. 10.1016/j.fertnstert.2021.09.014 34674825

[4] Kiviat NB Wølner-Hanssen P Eschenbach DA Wasserheit JN Paavonen JA Bell TA Endometrial histopathology in patients with culture-proved upper genital tract infection and laparoscopically diagnosed acute salpingitis. Am J Surg Pathol. (1990) 14(2):167–75. 10.1097/00000478-199002000-00008 2137304

[5] Espinós JJ Fabregues F Fontes J García-Velasco JA Llácer J Requena A Impact of chronic endometritis in infertility: a SWOT analysis. Reproduct BioMed. (2021) 42(5):939–51. 10.1016/j.rbmo.2021.02.003 33736994

[6] Kitaya K Ishikawa T. Chronic endometritis: simple can be harder than complex? Fertil Steril. (2021) 115(6):1443–4. 10.1016/j.fertnstert.2021.03.023 33892957

[7] Cao L Gao S Liu J Wang J Qin R. Selenomethionine protects against E. coli -induced endometritis by inhibiting inflammation and necroptosis via regulating the PPAR-γ/NF-κB pathway. Chem-Biol Interact. (2023) 379:110532. 10.1016/j.cbi.2023.110532 37150495

[8] Kimura F Takebayashi A Ishida M Nakamura A Kitazawa J Morimune A Review: chronic endometritis and its effect on reproduction. J Obstet Gynaecol Res. (2019) 45(5):951–60. 10.1111/jog.13937 30843321

[9] Sun T Jackson S Haycock JW MacNeil S. Culture of skin cells in 3D rather than 2D improves their ability to survive exposure to cytotoxic agents. J Biotechnol. (2006) 122(3):372–81. 10.1016/j.jbiotec.2005.12.021 16446003

[10] Psilopatis I Kokkali S Palamaris K Digklia A Vrettou K Theocharis S. Organoids: a new chapter in sarcoma diagnosis and treatment. Int J Mol Sci. (2022) 23(19):11271. 10.3390/ijms231911271 36232574 PMC9570355

[11] Tang S Parks SE Liao Z Cope DI Blutt SE Monsivais D. Establishing 3D endometrial organoids from the mouse uterus. J Vis Exp. (2023) (191). 10.3791/64448 PMC10208800 36688555

[12] Turco MY Gardner L Hughes J Cindrova-Davies T Gomez MJ Farrell L Long-term, hormone-responsive organoid cultures of human endometrium in a chemically defined medium. Nat Cell Biol. (2017) 19(5):568–77. 10.1038/ncb3516 28394884 PMC5410172

[13] Mannelli C Ietta F Avanzati AM Skarzynski D Paulesu L. Biological tools to study the effects of environmental contaminants at the feto–maternal interface. Dose Response. (2015) 13(4):1559325815611902. 10.1177/1559325815611902 26740808 PMC4679191

[14] Xu Y Hu J Lv Q Shi C Qiu M Xie L Endometrium-derived mesenchymal stem cells suppress progression of endometrial cancer via the DKK1-wnt/β-catenin signaling pathway. Stem Cell Res Ther. (2023) 14:159. 10.1186/s13287-023-03387-4 37287079 PMC10249217

[15] Knowlton AA Lee AR. Estrogen and the cardiovascular system. Pharmacol Ther. (2012) 135(1):54–70. 10.1016/j.pharmthera.2012.03.007 22484805 PMC5688223

[16] Baker JM Chase DM Herbst-Kralovetz MM. Uterine microbiota: residents, tourists, or invaders? Front Immunol. (2018) 9:208. 10.3389/fimmu.2018.00208 29552006 PMC5840171

[17] Molina NM Sola-Leyva A Haahr T Aghajanova L Laudanski P Castilla JA Analysing endometrial microbiome: methodological considerations and recommendations for good practice. Hum Reprod. (2021) 36(4):859–79. 10.1093/humrep/deab009 33532852

[18] Wang J Li Z Ma X Du L Jia Z Cui X Translocation of vaginal microbiota is involved in impairment and protection of uterine health. Nat Commun. (2021) 12:4191. 10.1038/s41467-021-24516-8 34234149 PMC8263591

[19] Moreno I Cicinelli E Garcia-Grau I Gonzalez-Monfort M Bau D Vilella F The diagnosis of chronic endometritis in infertile asymptomatic women: a comparative study of histology, microbial cultures, hysteroscopy, and molecular microbiology. Am J Obstet Gynecol. (2018) 218(6):602.e1–6. 10.1016/j.ajog.2018.02.012 29477653

[20] Murtinger M Wirleitner B Spitzer D Bralo H Miglar S Schuff M. Diagnosing chronic endometritis: when simplification fails to clarify. Hum Reprod Open. (2022) 2022(3):hoac023. 10.1093/hropen/hoac023 35722504 PMC9202642

[21] McQueen DB Perfetto CO Hazard FK Lathi RB. Pregnancy outcomes in women with chronic endometritis and recurrent pregnancy loss. Fertil Steril. (2015) 104(4):927–31. 10.1016/j.fertnstert.2015.06.044 26207958

[22] Kitaya K Matsubayashi H Yamaguchi K Nishiyama R Takaya Y Ishikawa T Chronic endometritis: potential cause of infertility and obstetric and neonatal complications. Am J Reprod Immunol. (2016) 75(1):13–22. 10.1111/aji.12438 26478517

[23] Letarov AV. Bacterial virus forcing of bacterial O-antigen shields: lessons from coliphages. Int J Mol Sci. (2023) 24(24):17390. 10.3390/ijms242417390 38139217 PMC10743462

[24] Xie X Chen X Zhang S Liu J Zhang W Cao Y. Neutralizing gut-derived lipopolysaccharide as a novel therapeutic strategy for severe leptospirosis. eLife. (2024) 13:RP96065. 10.7554/eLife.96065.3 38818711 PMC11142641

[25] Kyo S Nakamura M Kiyono T Maida Y Kanaya T Tanaka M Successful immortalization of endometrial glandular cells with normal structural and functional characteristics. Am J Pathol. (2003) 163(6):2259–69. 10.1016/S0002-9440(10)63583-3 14633600 PMC1892381

[26] Cooke PS Buchanan DL Young P Setiawan T Brody J Korach KS Stromal estrogen receptors mediate mitogenic effects of estradiol on uterine epithelium. Proc Natl Acad Sci U S A. (1997) 94(12):6535–40. 10.1073/pnas.94.12.6535 9177253 PMC21085

[27] Simintiras CA Dhakal P Ranjit C Fitzgerald HC Balboula AZ Spencer TE. Capture and metabolomic analysis of the human endometrial epithelial organoid secretome. Proc Natl Acad Sci U S A. (2021) 118(15):e2026804118. 10.1073/pnas.2026804118 33876774 PMC8053979

[28] Kakni P López-Iglesias C Truckenmüller R Habibović P Giselbrecht S. PSC-derived intestinal organoids with apical-out orientation as a tool to study nutrient uptake, drug absorption and metabolism. Front Mol Biosci. (2023) 10:1102209. 10.3389/fmolb.2023.1102209 36743212 PMC9889654

[29] Sette G Lo Cicero S Blaconà G Pierandrei S Bruno SM Salvati V Theratyping cystic fibrosis in vitro in ALI culture and organoid models generated from patient-derived nasal epithelial conditionally reprogrammed stem cells. Eur Respir J. (2021) 58(6):2100908. 10.1183/13993003.00908-2021 34413153 PMC8675295

[30] Wijesekara P Patel KZ Otto EL Campbell PG Ren X. Protocol to engineer apical-out airway organoids using suspension culture of human airway basal stem cell aggregates. STAR Protocols. (2023) 4(2):102154. 10.1016/j.xpro.2023.102154 36917607 PMC10025264

[31] Silva S Bicker J Falcão A Fortuna A. Air-liquid interface (ALI) impact on different respiratory cell cultures. Eur J Pharm Biopharm. (2023) 184:62–82. 10.1016/j.ejpb.2023.01.013 36696943

[32] Prescott RA Pankow AP de Vries M Crosse KM Patel RS Alu M A comparative study of in vitro air–liquid interface culture models of the human airway epithelium evaluating cellular heterogeneity and gene expression at single cell resolution. Respir Res. (2023) 24:213. 10.1186/s12931-023-02514-2 37635251 PMC10464153

[33] Fitzgerald HC Dhakal P Behura SK Schust DJ Spencer TE. Self-renewing endometrial epithelial organoids of the human uterus. Proc Natl Acad Sci U S A. (2019) 116(46):23132–42. 10.1073/pnas.1915389116 31666317 PMC6859318

[34] Seppälä M Koistinen H Glycodelins KR. Trends Endocrinol Metabol. (2001) 12(3):111–7. 10.1016/S1043-2760(00)00365-9 11306335

[35] Yue X Wu L Hu W. The regulation of leukemia inhibitory factor. Cancer Cell Microenviron. (2015) 2(3):e877. 10.14800/ccm.877 26807429 PMC4722946

[36] Rosario GX Stewart CL. The multifaceted actions of leukaemia inhibitory factor in mediating uterine receptivity and embryo implantation. Am J Reprod Immunol. (2016) 75(3):246–55. 10.1111/aji.12474 26817565

[37] Hambartsoumian E. Endometrial leukemia inhibitory factor (LIF) as a possible cause of unexplained infertility and multiple failures of implantation. Am J Reprod Immunol. (1998) 39(2):137–43. 10.1111/j.1600-0897.1998.tb00345.x 9506211

[38] Peyghambari F Salehnia M Forouzandeh Moghadam M Rezazadeh Valujerdi M Hajizadeh E. The correlation between the endometrial integrins and osteopontin expression with pinopodes development in ovariectomized mice in response to exogenous steroids hormones. Iran Biomed J. (2010) 14(3):109–19. 21079662 PMC3904062

[39] Lai ZZ Wang Y Zhou WJ Liang Z Shi JW Yang HL Single-cell transcriptome profiling of the human endometrium of patients with recurrent implantation failure. Theranostics. (2022) 12(15):6527–47. 10.7150/thno.74053 36185612 PMC9516226

[40] Rettberg JR Yao J Brinton RD. Estrogen: a master regulator of bioenergetic systems in the brain and body. Front Neuroendocrinol. (2014) 35(1):8–30. 10.1016/j.yfrne.2013.08.001 23994581 PMC4024050

[41] Tsuchiya Y Nakajima M Yokoi T. Cytochrome P450-mediated metabolism of estrogens and its regulation in human. Cancer Lett. (2005) 227(2):115–24. 10.1016/j.canlet.2004.10.007 16112414

[42] Nilsson S Mäkelä S Treuter E Tujague M Thomsen J Andersson G Mechanisms of estrogen action. Physiol Rev. (2001) 81(4):1535–65. 10.1152/physrev.2001.81.4.1535 11581496

[43] Patel S Homaei A Raju AB Meher BR. Estrogen: the necessary evil for human health, and ways to tame it. Biomed Pharmacother. (2018) 102:403–11. 10.1016/j.biopha.2018.03.078 29573619

[44] Lien WH Fuchs E. Wnt some lose some: transcriptional governance of stem cells by wnt/β-catenin signaling. Genes Dev. (2014) 28(14):1517–32. 10.1101/gad.244772.114 25030692 PMC4102759

[45] Nusse R Clevers H. Wnt/β-catenin signaling, disease, and emerging therapeutic modalities. Cell. (2017) 169(6):985–99. 10.1016/j.cell.2017.05.016 28575679

[46] Merenda A Fenderico N Maurice MM. Wnt signaling in 3D: recent advances in the applications of intestinal organoids. Trends Cell Biol. (2020) 30(1):60–73. 10.1016/j.tcb.2019.10.003 31718893

[47] Liu S Fan W Gao X Huang K Ding C Ma G Estrogen receptor alpha regulates the wnt/β-catenin signaling pathway in colon cancer by targeting the NOD-like receptors. Cell Signal. (2019) 61:86–92. 10.1016/j.cellsig.2019.05.009 31121307

[48] Kattoor AJ Pothineni NVK Palagiri D Mehta JL. Oxidative stress in atherosclerosis. Curr Atheroscler Rep. (2017) 19(11):42. 10.1007/s11883-017-0678-6 28921056

[49] Teoh JP Li X Simoncini T Zhu D Fu X. Estrogen-mediated gaseous signaling molecules in cardiovascular disease. Trends Endocrinol Metab. (2020) 31(10):773–84. 10.1016/j.tem.2020.06.001 32682630

